# Unveiling Opportunities for Intervention: A Prospective Cohort Study Investigating the Clinical Significance of Diffusion-Weighted Imaging (DWI)-Fluid-Attenuated Inversion Recovery (FLAIR) Mismatch Beyond the Window Period in Acute Ischemic Stroke

**DOI:** 10.7759/cureus.63996

**Published:** 2024-07-06

**Authors:** Rangaramanujanaidu Ravichandran, Vasanthi N, Nayyar Iqbal

**Affiliations:** 1 General Medicine, Pondicherry Institute of Medical Sciences, Pondicherry, IND; 2 General Medicine/Endocrinology, Pondicherry Institute of Medical Sciences, Pondicherry, IND

**Keywords:** mrs, nihss, clinical outcomes, thrombolysis, dwi-flair mismatch, acute ischemic stroke

## Abstract

Introduction: Acute ischemic stroke causes irreversible damage to the brain parenchyma surrounded by salvageable tissue known as the ischemic penumbra. Magnetic resonance imaging (MRI), particularly the mismatch between abnormal diffusion-weighted imaging (DWI) signals and normal fluid-attenuated inversion recovery (FLAIR) signals, plays a critical role in detecting ischemic penumbra. It also allows for the identification of patients who may benefit from reperfusion therapy. Hence, this prospective cohort study aimed to explore the correlation between DWI-FLAIR mismatch and clinical outcomes in acute ischemic stroke patients, specifically those with delayed or uncertain symptom onset, offering potential insights into reperfusion therapy.

Methodology: A total of 38 thrombotic stroke patients aged above 18 were included in this prospective cohort study. Baseline data, including demographics, lifestyle factors, and medical history, were recorded. DWI-FLAIR mismatch was evaluated through brain MRI within 4.5 hours to 12 hours of symptom onset.

Results: Of the cohort, 63.2% were males, predominantly in the 61-70 age group. Smoking and alcohol consumption were reported by 15.79% each. DWI-FLAIR mismatch was present in 20 out of 38 subjects. No statistically significant differences were noted in the mean National Institutes of Health Stroke Scale (NIHSS) and Modified Rankin Scale (MRS) scores between subjects with and without DWI-FLAIR mismatch. Thrombolysis in wake-up stroke subjects demonstrated a substantial reduction in mean MRS at discharge (1.29±0.95) and at six to eight weeks (1.71±1.11), suggesting potential benefits on functional outcomes.

Conclusion: The prevalence of DWI-FLAIR mismatch was seen in the majority of patients beyond their window period and also showed beneficiary outcomes with a mean reduction in NHISS and MRS scores following thrombolysis.

## Introduction

Stroke, ranking as the second leading global cause of death, claimed 5.87 million lives in 2010, with a staggering 85% of the burden falling on low- and middle-income countries (LMICs) according to the Global Burden of Disease (GBD) reports of 2001 [1,2]. Studies reveal a doubling of stroke incidence in LMICs, coupled with a 42% decline in high-income countries [3]. In India, stroke is a prominent cause of death and disability, with an estimated prevalence of 105-152 per 100,000 persons annually over the past decade. Incidence rates range from 119 to 145 per 100,000 based on recent population-based studies [3,4].

Approximately, 87% of all strokes are ischemic, with intravenous thrombolytic treatment being the sole FDA-approved pharmacological option, albeit limited in accessibility [5]. The acute phase of stroke results in an irreversibly damaged ischemic core alongside salvageable surrounding tissue, known as the ischemic penumbra. This penumbra, defined by Symon, Lassen, and colleagues in the 1970s, represents brain tissue with insufficient blood flow to maintain neural electric activity but enough to preserve ion channel function [6].

Ischemic penumbra is a dynamic process which exists for a short period of time. This core area is subjected to a wave of deleterious metabolic processes from where irreversible necrosis propagates to the neighbouring tissue over time and leads to worsening clinical outcomes. It was shown that, if blood flow could be restored to this area, the tissue could survive and function again, and growth of the core could be prevented. The penumbra thus became the target for all acute stroke interventions - to preserve the viability of the tissue and restore function. However, the time window of therapeutic opportunity is variable and ill-defined; it may take very short to several hours in the moderately ischemic surrounding tissue to turn to irreversible damage [7,8].

Effective MR imaging techniques sensitive to areas at risk for infarction are crucial for the therapeutic window of cerebral ischemia [9]. Diffusion-weighted imaging (DWI) and fLuid-attenuated inversion recovery (FLAIR) are widely used magnetic resonance imaging (MRI) modalities for identifying the ischemic penumbra. The present study aims to illustrate a mismatch between abnormal DWI signals and normal FLAIR on brain MRI in acute ischemic stroke cases with delayed or uncertain symptom onset, allowing for reperfusion therapy.

The primary objective of this study was to determine the proportion of patients presenting with wake-up stroke or those who present between four and 12 hours after the onset of thrombotic stroke who show DWI-FLAIR mismatch. The secondary objectives were to determine the association of DWI-FLAIR mismatch and other clinical determinants in response to thrombolytic therapy as defined by a modified Rankin scale at discharge and 6-12 weeks follow-up and to identify the clinical predictors of response to thrombolysis in the group who have undergone thrombolysis as a treatment for thrombotic stroke.

## Materials and methods

This prospective cohort study was done to investigate the clinical significance of ischemic penumbra in stroke in the Department of General Medicine, Pondicherry Institute of Medical Sciences, between November 2019 and October 2021.

Those patients who presented with thrombotic stroke and aged above 18 years, encompassing both those experiencing a 'wake stroke'; patients with delayed symptom onset within the 4.5-12-hour timeframe; those with confirmed infarcts on CT scans; those showing focal neurological deficits; and those who were scheduled for an MRI as part of routine procedures were included. We excluded patients with contraindications for MRI, such as cardiac pacemakers, orthopaedic implants, artificial heart valves, joint replacements, brain aneurysmal clips, intrauterine devices (IUDs), and cochlear implants; those with cardioembolic strokes; and those originating from the posterior circulation.

Based on the previous study done by Khatibi et al. [10], which reported an approximate prevalence of 40% for the identified MRI findings, we determined that a sample size of 92 subjects with ischemic stroke would afford us the ability to achieve this estimation with a precision of 10% and a confidence level of 95%. The sample size was increased to 100. However, the study faced limitations due to unmet sample size targets amid COVID-19, affecting precision. An interventional design was impractical, necessitating the use of DWI-FLAIR prevalence as a surrogate. After getting Institutional Ethics Committee (IEC: RC/19/84) consent, the sample size was reduced to 38.

All patients were enrolled in the study after obtaining informed consent and approval to participate. Patients presenting to the Emergency Department and General Medicine Outpatient Department were assessed through a rigorous eligibility screening process. Patients who belonged to the wake-up stroke group underwent a CT brain to rule out any haemorrhages followed by an MRI brain. In the MRI brain, we looked for DWI and FLAIR mismatch. The other group is those patients who presented after their window period (4.5 hours). These patients also received a similar assessment. In both groups, we assessed how many patients had mismatches in MRI. Those patients who were found to have mismatch were treated with thrombolytic therapy, and their outcomes were measured using the NIHSS and MRS scores. Emphasis was placed on tracking clinical status at both discharge and during follow-up visits at 6-8 weeks post-discharge. To mitigate potential errors, pertinent data were systematically gathered utilizing a standardized proforma. Subsequently, these data were meticulously transcribed into an Excel spreadsheet and securely stored within a confidential database.

For statistical analysis, all data, systematically recorded in a Microsoft Excel spreadsheet, underwent comprehensive analysis utilizing the Statistical Product and Service Solutions (SPSS, version 21; IBM SPSS Statistics for Windows, Armonk, NY) software. Normality assessments were conducted employing both the Kolmogorov-Smirnov and Shapiro-Wilk tests. Descriptive statistics were subsequently computed to provide a comprehensive overview of the dataset. Independent samples t-test was employed for the comparison of continuous variables, specifically distinguishing patients with positive findings in DWI and FLAIR from those with negative results in both modalities. Categorical variables were subjected to analysis using the chi-square test. The threshold for statistical significance was established at a p-value below 0.05.

## Results

We enrolled 38 patients which included the wake-up stroke group and those that crossed the window period. All patients were assessed for DWI and FLAIR mismatch.

Table 1 outlines baseline demographic characteristics, revealing that 60.53% were males. Age distribution showed 10.53% aged <50 years, 28.95% between 51 and 60 years, 44.74% between 61 and 70 years, and 15.79% between 71 and 80 years. Lifestyle factors included 15.79% reporting smoking habits, and an equal proportion were habituated to regular alcohol consumption.

**Table 1:** Demographic and Clinical Characteristics of Study Participants CAD - coronary artery disease

Parameter		Frequency	Percentage
Gender	Male	23	60.53
	Female	15	39.47
Age	≤ 50 Years	4	10.53
	51-60 years	11	28.95
	61-70 years	17	44.74
	71-80 years	6	15.79
Smoking habits	No	32	84.21
	Yes	6	15.79
Alcohol consumption	No	32	84.21
	Yes	6	15.79
Diabetes	No	12	31.58
	Yes	26	68.42
Hypertension	No	13	34.21
	Yes	25	65.79
CAD	No	28	73.68
	Yes	10	26.32

Table 2 delves into the association between stroke type, onset time, and DWI-FLAIR mismatch. Among the 38 subjects, 20 exhibited DWI-FLAIR mismatch, with 72.73% experiencing non-wakeup strokes. Conversely, of the 18 subjects without DWI-FLAIR mismatch, 55.56% had wake-up strokes. 

**Table 2:** Association Between the Type of Stroke and DWI-FLAIR Mismatch DWI - Diffusion-weighted imaging, FLAIR - Fluid-attenuated inversion recovery

Condition		DWI-FLAIR mismatch		Total n (%)	P value
		No, n (%)	Yes, n (%)		
Type of stroke	Non-wakeup	3 (27.27)	8 (72.73)	11 (100)	0.16
	Wakeup	15 (55.56)	12 (44.44)	27 (100)	

Table 3 compares mean changes in NIHSS and MRS scores with and without DWI-FLAIR mismatch. No statistically significant differences were found, indicating comparable outcomes between the groups.

**Table 3:** Comparative Analysis of Neurological Outcome Measures Based on the Presence of DWI-FLAIR Mismatch NIHSS - National Institute of Health Stroke Scale, DWI - Diffusion-weighted imaging, FLAIR- Fluid-attenuated inversion recovery, MRS - Modified Rankin Scale

Parameter	DWI-FLAIR mismatch	N	Mean	Std. deviation	P value
Change in NIHSS score from baseline to discharge	No	18	0.83	6.61	0.23
	Yes	20	1.65	4.30	
Change in NIHSS score from baseline to 6-8 weeks after discharge	No	18	4.76	4.48	0.16
	Yes	20	3.82	5.50	
Change in MRS score from baseline to discharge	No	18	0.39	0.70	0.55
	Yes	20	0.41	1.00	
Change in MRS score from baseline to 6-8 weeks after discharge	No	18	0.83	1.10	0.94
	Yes	20	1.18	1.42	

Table 4 focuses on patients undergoing thrombolysis, revealing a notable reduction in mean MRS for wake-up stroke subjects (1.29±0.95) compared to non-wake-up stroke subjects (0.33±1.15) from admission to discharge. At six to eight weeks, wake-up stroke subjects who underwent thrombolysis showed a mean MRS reduction of 1.71±1.11, while non-wake-up stroke subjects exhibited a reduction of 0±1.73. The difference in the mean NIHSS score at six to eight weeks highlighted a higher reduction for wake-up stroke subjects undergoing thrombolysis (9.14±4.53) compared to non-wake-up stroke subjects (7±2.65). These findings emphasize the potential impact of thrombolysis on functional outcomes, particularly in the context of wake-up strokes.

**Table 4:** Comparative Analysis of Neurological Outcome Measures Based on the Type of Stroke NIHSS - National Institute of Health Stroke Scale, MRS - Modified Rankin Scale

Parameter	Type of stroke	N	Mean	Std. deviation	P value
Change in NIHSS score from baseline to discharge	Wakeup	7	4.29	4.07	0.83
	Non-wakeup	3	-4.00	16.46	
Change in NIHSS score from baseline to 6-8 weeks after discharge	Wakeup	7	9.14	4.53	0.38
	Non-wakeup	3	7.00	2.65	
Change in MRS score from baseline to discharge	Wakeup	18	1.29	0.95	0.38
	Non-wakeup	20	0.33	1.15	
Change in MRS score from baseline to 6-8 weeks after discharge	Wakeup	18	1.71	1.11	0.18
	Non-wakeup	20	0.00	1.73	

## Discussion

Stroke is the world's second biggest cause of mortality, with an estimated 6.5 million fatalities and 113 million disability-adjusted life years (DALY) in 2013. A multitude of clinical evidence has demonstrated the safety and effectiveness of IV thrombolysis in suitable patients throughout the last few decades. Early treatment, followed by the screening of eligible candidates for thrombolysis, is critical for effective thrombolytic therapy. Clinical trials show that using imaging to rule out bleeding in stroke patients before starting IV thrombolytic treatment is a good idea. It has two purposes: first, to diagnose or confirm the presence of a stroke, and, second, to assess the quantity of possibly salvageable brain tissue and permanently infarcted tissue; both are required, the first for management strategy planning and the second for prognostication.

In our study, 63.2% of the study subjects were males and the remaining 36.8% were females. Similar results were observed in the study conducted by Kamalakannan et al. and Thomalla et al. [11,12], which showed a higher prevalence among males than females. Aging population dynamics were evident, with the majority in the 61-70 years age group. A study done by Rodríguez-Campello et al. [13] showed an increased prevalence of smoking habits among subjects with ischemic penumbra. In our study, the prevalence of smoking and alcohol consumption was 15.76% and 15.79%, respectively, which may be because of population variability. Six out of 38 (15.79%) of our study subjects did not have any comorbidities; on the other hand, 68.42% of the subjects were found to be diabetic, 65.79% of the subjects were found to be hypertensive, and 26.31% subjects were found to have coronary artery disease (CAD). Similar results were found in the study done by Rodríguez-Campello et al. [13] in which hypertension was the most observed comorbidity, followed by diabetes and CAD. The study also showed that the association between diabetes, CAD, and penumbra was significant; on the other hand, the association between hypertension and penumbra was not significant.

A study done by Odland et al. [14] showed a mean reduction in the NIHSS of 4.8 among patients who had DWI-FLAIR mismatch and received intravenous thrombosis. Similarly, a study done by Madai et al. [15] showed high NIHSS scores before treatment. Similar results were seen in our study where the mean changea in NIHSS and MRS scores at the time of discharge from that at admission among those who had DWI-FLAIR mismatch were 1.65±430 and 0.41±1.0 and NIHSS and MRS scores of 0.83±6.61 and 0.39±0.70, respectively, among those who did not have DWI-FLAIR mismatch. The mean change in NIHSS and MRS score at six to eight weeks after discharge from that at admission among those who had DWI-FLAIR mismatch was 3.82±5.50 and 1.18±1.42 as against NIHSS and MRS scores of 4.76±4.48 and 0.83±1.10, respectively, among those who did not have DWI-FLAIR mismatch. However, the differences were not significant among subjects with DWI FLAIR mismatch and subjects without DWI FLAIR mismatch. In our study, from the time of admission to six to eight weeks after discharge, there was a reduction in mean NIHSS and MRS scores of 3.90±4.84 and 1.10±1.20 among non-wakeup stroke as compared to 4.46±5.11 and 0.96±1.31, respectively, among wake-up stroke. Similar results were found in a study done by Odland et al. [14] in which patients showed 2 points drop in MRS scores after six months.

Limitation

Since the study is single-centred and non-probability convenience sampling was adopted, the study lacked the scientific rigor and external validity required to support widespread changes in practice and must be considered as supportive of existing policy regarding using DWI-FLAIR as an indication for thrombolysis in patients in wake-up stroke and those presenting between 4.5 hours and 12 hours of onset.

## Conclusions

The prevalence of DWI-FLAIR mismatch was seen in the majority of patients beyond their window period and showed beneficiary outcomes with a mean reduction in NHISS and MRS scores following thrombolysis.
